# Effect of water temperature on the morbidity of *Tetracapsuloides bryosalmonae* (Myxozoa) to brown trout (*Salmo trutta*) under laboratory conditions

**DOI:** 10.1111/jfd.13361

**Published:** 2021-03-03

**Authors:** Karoline Waldner, Martin Borkovec, Florian Borgwardt, Günther Unfer, Mansour El‐Matbouli

**Affiliations:** ^1^ Clinical Division for Fish Medicine University of Veterinary Medicine Vienna Austria; ^2^ Bandgasse 33‐41/2/15 Vienna Austria; ^3^ University of Natural Resources and Life Sciences Vienna, Institute of Hydrobiology and Aquatic Ecosystem Management Vienna Austria

**Keywords:** brown trout, clinical signs, kidney swelling, PKD, survival, water temperature

## Abstract

Proliferative kidney disease (PKD) is a disease found in salmonid fish that is widespread in Europe and North America. The dependency of the clinical signs on the water temperature is extensively reported in rainbow trout, but detailed information on brown trout is lacking. In this study, juvenile brown trout were exposed to the spores of *Tetracapsuloides bryosalmonae* and then kept at different ambient water temperatures (16°C, 19°C and 22°C) for 10 weeks along with recording of morbidity throughout the experiment. At 6, 8 and 10 weeks post‐exposure, fish from each temperature group were sampled and underwent pathoanatomical examination to survey disease progression. At 16°C, brown trout showed a significantly higher survival probability compared to those kept in 19°C and 22°C water. Additionally, the parasitic burden (MSQ) was higher and the clinical signs were more pronounced in the brown trout kept at 19°C and 22°C compared with the ones kept at 16°C. This study highlights the correlation of PKD outbreaks and water temperature increases related to global climate change, which will impact the future distribution of brown trout in natural waters.

## 1 INTRODUCTION

Proliferative kidney disease (PKD) is a parasitic disease of salmonid fish with a complex life cycle (Okamura et al., 2015). The causative pathogen for this disease is *Tetracapsuloides bryosalmonae* (Myxozoa: Malacosporea). Its life cycle alternates between bryozoans as main hosts and salmonid fish as intermediate hosts (Anderson et al., 1999). Firstly, the spores are released by the infected bryozoan species, such as *Cristatella mucedo*, *Pectinatella magnifica*, *Plumatella rugosa*, *Plumatella emarginata* and mainly *Fredericella sultana* (Bryozoa: Phylactolaemata) (Hartikainen et al., 2014; Longshaw et al., 1999), and enter the fish host through the gills and the skin (Grabner & El‐Matbouli, 2010; Longshaw et al., 2002). After invasion, the parasite migrates to the target organ kidney through the bloodstream, where it causes inflammation, eventually leading to an enlargement of the kidney (Clifton‐Hadley et al., 1987; Hedrick et al., 1993; Holzer et al., 2006). In brown trout (*Salmo trutta;* Linnaeus, 1758) and brook trout (*Salvelinus fontinalis*; Mitchill, 1814), the parasite forms intraluminal sporogonic stages that are excreted via the urine for further transmission to bryozoan colonies (Hedrick et al., 2004; Grabner & El‐Matbouli, 2008). The parasite development in the bryozoan host is positively correlated to temperature and nutrient content of the water (El‐Matbouli & Hoffman, 2002; Hartikainen et al., 2009; Tops et al., ,^2006^, ^2009^). In natural water bodies, the abundance of spore‐producing *Fredericella sultana* increases with the distance to the spring and depends more on mean elevation, local slope and the presence of moraines than on water temperature (Carraro et al., 2017, 2018).

Similar to most fish diseases like Columnaris, Ichthyophthiriasis or KHV (Gilad et al., 2003; Karvonen et al., 2010), PKD is also highly temperature‐dependent as the immune system of fish is impacted by the surrounding water temperature (Ferguson & Needham, 1978; Le Morvan et al., 1998). In rainbow trout, the onset of clinical signs begins at 10°C (Palikova et al., 2017), and the severity of the disease increases with increasing water temperature. The cumulative mortality is 5.6% at 12°C and can reach up to 85% within 49 days post‐exposure at 19°C (Bettge et al., 2009; Bettge et al., 2009). However, only little information is available regarding brown trout. A couple of animal experiments, under laboratory conditions, were performed on this species investigating the response to *T*. *bryosalmonae*. No significant differences in cumulative mortality rates were observed between exposed and control brown trout kept for 1 year at 12°C and 15°C (Strepparava et al., ,^2018^, ^2020^). In another study, fish were exposed to a low number of *T. bryosalmonae* spores (1 * 10^5^ ± 3 * 10^4^ DNA copies per fish) for a short period of time (1 hr); however, no conclusion about the mortality rates of the PKD‐infected fish could be made (Bailey et al., 2017). In brown trout exposed to a PKD endemic river in a cage where water temperature exceeded 15°C for 38 days in a row, disease‐associated mortality was 15% (Schmidt‐Posthaus et al., 2015). Wild brown trout populations may be even more severely affected by PKD‐induced anaemia, resulting in reduced aerobic scope and lowered upper thermal tolerance, thereby decreasing survival of brown trout, especially in smaller juvenile fish (Bruneaux et al., 2017; Debes et al., 2017; Stauffer et al., 2017). However, very little is known about the PKD‐linked mortality rates in the natural environment (Ahmad et al., 2020; Bailey et al., 2018).

A variety of factors is putting wild brown trout population under pressure, including man‐made changes like the missing changes in the water discharges, channelization of rivers, interruption of stream connectivity, water pollution, removal of riparian vegetation and management methods such as fish stocking as well as returning predators (Almodóvar et al., 2012; Borgwardt et al., 2020). Climate change is one of them already causing a loss of suitable thermal habitat in the lower distribution range of brown trout (Almodóvar et al., 2012) and will continue to progress as the global surface temperature is expected to rise by 1.5°C above the preindustrial levels till 2050 (IPCC, 2018). Mountain streams are considered to remain suitable habitats for cold water fish like brown trout (Isaak et al., 2016), but the Austrian Panel on Climate Change (APCC) (Österreichischer Sachstandsbericht Klimawandel 2018) expects that the warming in the alpine area could be the same as the European average or even above (Kromp‐Kolb et al., 2014), which is already affecting the water temperature in Austrian rivers and also the species living in the aquatic environment (Markovic et al., 2013; Pletterbauer et al., 2016). Water temperature is an essential parameter in riverine ecosystems, especially for temperature‐sensitive species like brown trout that have an optimal temperature range between 4 –19°C (Elliott & Elliott, 2010).

Proliferative kidney disease ‐infected salmonid fish were reported all across Europe, from Italy to Norway and Iceland (Beraldo et al., 2006; Jencic et al., 2014; Kristmundsson et al., 2010; Peeler et al., 2008) In Switzerland, PKD was considered one of the main reasons for declining wild brown trout populations (Wahli et al., 2007). In Austria, PKD was first detected in the river Kamp in 2014 (Unfer et al., 2015), and later, many PKD cases were confirmed in Austrian rivers (Lewisch et al., 2018; Waldner et al., 2019).

The aim of this study is the validation of the thermal effects on morbidity in brown trout. Accordingly, brown trout were exposed to *T. bryosalmonae* at three different water temperatures (16°C, 19°C and 22°C) for 10 weeks. The hypothesis was that increasing water temperature will increase both clinical signs and morbidity rates of PKD‐infected fish.

## 2 MATERIAL AND METHODS

### 2.1 Fish

Brown trout (*n* = 294, size: 5.8 ± 0.4 cm, weight: 2.3 ± 0.6 g) were purchased from a fish farm, where fish were supplied by spring water with no PKD history. After an acclimatization period of 2 weeks and a general health examination including skin and gill smear as well as a real‐time PCR targeting *T. bryosalmonae* to rule out a pre‐existing PKD infection, the fish were randomly assigned to different temperature groups and allocated to 75‐L tanks with water temperature gradually increasing 1°C per day until the final temperature was reached.

### 2.2 Experimental design

The desired water temperature for all the temperature groups, that is 16°C, 19°C and 22°C, respectively, was maintained as described previously by Kumar et al. (2013). Infection has been performed as previously described by Kumar et al. (2013) using spore sacs gained from laboratory‐cultivated *Fredericella sultana* colonies. For the infection, all 60 brown trout of each temperature group were put together in one tank and infected using 35 spore sacs of similar size. During infection, the water flow was stopped, and the water temperature was maintained at 16°C, 19°C and 22°C, respectively. After 1 day of exposure, brown trout from each temperature group were randomly split up into triplicates of 20 fish per tank and maintained at the respective water temperature. For every temperature group, a control group having 38 brown trout was kept under the same conditions in duplicate (19 fish per tank). Brown trout were fed twice a day at the rate of 1% of their body weight (Aqua Dynamic Semi Swim/2; Garant Aqua), and oxygen levels of the water were closely monitored during the whole experiment. After 6, 8 and 10 weeks post‐exposure, nine randomly selected fish from every infected and control group were killed by sodium hydrogen carbonate‐buffered MS222 (1 g/L) and examined to monitor the progress of the disease. Further, fish were observed every 2 hr between 8 a.m. and 8 p.m. every day and scored according to a score sheet (Appendix S1). If a fish reached a score of 3 in one category or a total score higher than 8, it was killed to prevent unnecessary animal suffering. In this paper, we further refer to these fish as killed. Killed brown trout were counted as events while sampled fish were considered as censored data. For ethical reasons, no fish were allowed to die during the experiment. Therefore, we refer to morbidity instead of mortality throughout the text.

### 2.3 General pathoanatomical examination

All killed fish were subjected to a general pathoanatomical examination, including the testing of skin and gill smears. Recorded parameters were weight, length and the kidney swelling index (KSI) (Clifton‐Hadley et al., 1987). Since the spleen is another target organ of *T. bryosalmonae*, the spleen score ([a] no pathological changes, [b] low‐grade enlargement, [c] moderate enlargement and [d] high‐grade enlargement) was also recorded. Further, the bacteriological examination of the head kidney was performed on COS agar. In the case of bacterial growth, colonies were subcultured and investigated using MALDI‐TOF for species differentiation. A sample of the posterior kidney was preserved in RNA later at −20°C in 2‐ml tubes until DNA isolation was performed.

### 2.4 Detection and quantification of *T. bryosalmonae* in kidney tissue

The DNA was isolated from the tissue samples using DNeasy Blood and Tissue Kit (QIAGEN). The DNA concentration was then determined using Nanodrop to standardize the amount of fish tissue in the sample and thus allow comparison of the samples taken. RT‐qPCR was conducted as described in (Grabner & El‐Matbouli, 2009) using the primer pair, PKD‐real F and PKD‐real R, which produced a fragment of 166 bp. A final concentration of 700 ng of DNA per reaction was adjusted using PCR grade water, and the final volume of the reaction mix was 25 μl per reaction. Every sample was run in duplicates. A non‐template control was included in every run. In order to quantify the amount of *T. bryosalmonae* DNA in the samples, a standard curve was run in duplicate on every plate alongside with the samples. For the standard curve, the fragment was cloned using the primer pair 5F and 6R (435 bp) (Kent et al., 1998) into the PCR TOPO vector using the TOPO TA cloning kit. The plasmid was extracted using the QIAprep Spin Miniprep Kit (QIAGEN) and linearized enzymatically with NotI restriction enzyme. The product was cleaned up using the MinElute Gel Extraction Kit (QIAGEN) followed by measurement of DNA concentration using the NanoDrop spectrophotometer. The copy number was calculated by entering plasmid concentration and nucleotide length in http://cels.uri.edu/gsc/cndna.html. Later, a 10‐fold serial dilution from the plasmid stock was prepared using EB buffer (QIAGEN). All the qRT‐PCRs were run in the CFX96 Touch Real‐Time PCR Detection System thermocycler (Bio‐Rad) and were analysed using the Bio‐Rad CFX Maestro software (Bio‐Rad) to calculate the mean starting quantities (MSQ) of the parasitic DNA in the samples. Samples were excluded from analysis if the difference between the Cq values of the duplicates was greater than 1.

### 2.5 Statistics

All results were statistically analysed using R, version 4.0.2 (R Core Team, 2020). Plots were created using the package "ggplot2" (Wickham, 2016). *p*‐values were considered statistically significant when <.05. Treating all killed fish as events and all sampled fish as right‐censored samples, Kaplan–Meier curves were plotted for all temperature groups, and pairwise comparisons were calculated using the log‐rank test. Values of KSI, spleen score and MSQ underwent a descriptive analysis and were displayed for all killed brown trout using LOESS curves over the whole observation period and for all sampled fish using boxplots of the three samplings. Cumulative morbidity was calculated for each temperature group by dividing the number of fish killed due to clinical signs over the number of experimental fish subtracting the number of sampled fish.

## 3 RESULTS

### 3.1 Morbidity

**FIGURE 1:**
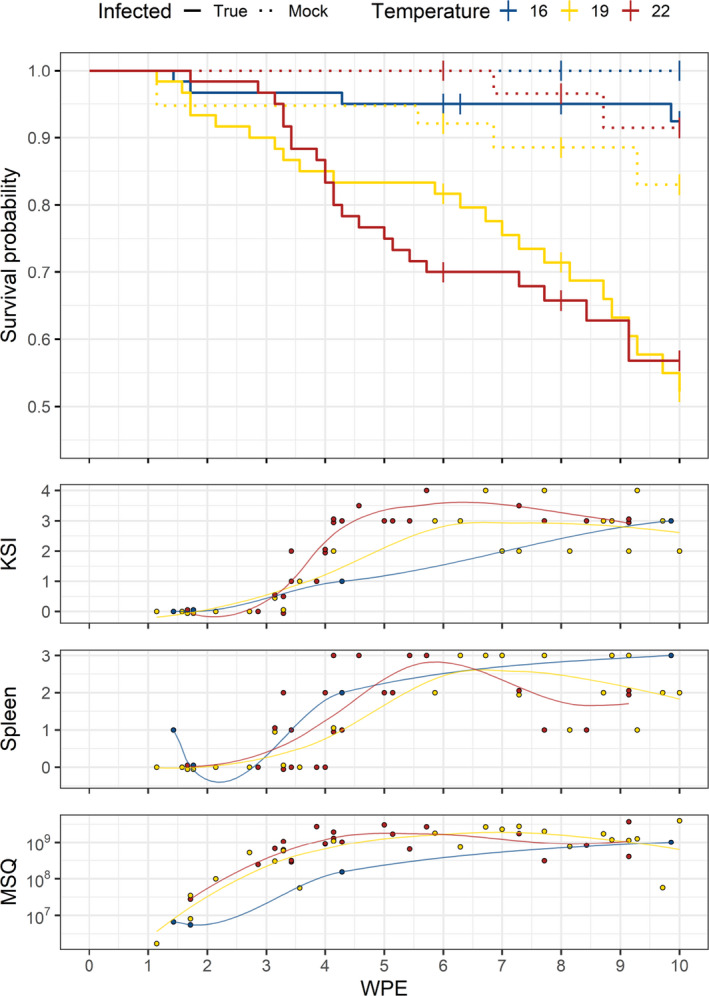
Plots of all the temperature groups showing survival probability, KSI, spleen score and mean starting quantity (MSQ) values of killed brown trout (Salmo trutta) after 10 weeks post‐exposure. Exposed groups are represented by a continuous line and control groups by dashed lines. Blue colour represents 16°C, yellow represents 19°C, and red indicates 22°C. Points denote killed fish [Colour figure can be viewed at wileyonlinelibrary.com]

At 16°C water temperature, 6 of the exposed brown trout were killed. Two out of these had to be put down because of a cannibalistic attack (attacker and victim) that occurred 44 days post‐exposure (dpe). These two fish were treated as censored data in the survival curve, shown in Figure 1. At 19°C, five control and 23 exposed fish were killed within the 10 weeks post‐exposure. At 22°C, two control and 23 exposed fish had to be killed. These results showed cumulative morbidity of 12.1% in the 16°C group, while cumulative morbidity in the 19°C and 22°C groups was 69.7%. Figure 1 shows the survival curve for all the exposed groups. Highly significant differences (*p* <.0001) were detected in the hazard functions of infected fish between the temperature group 16°C and the temperature groups 19°C and 22°C. No significant difference was observed in the hazard functions of exposed fish between the temperature groups 19°C and 22°C.

### 3.2 General pathoanatomical examination

**FIGURE 2:**
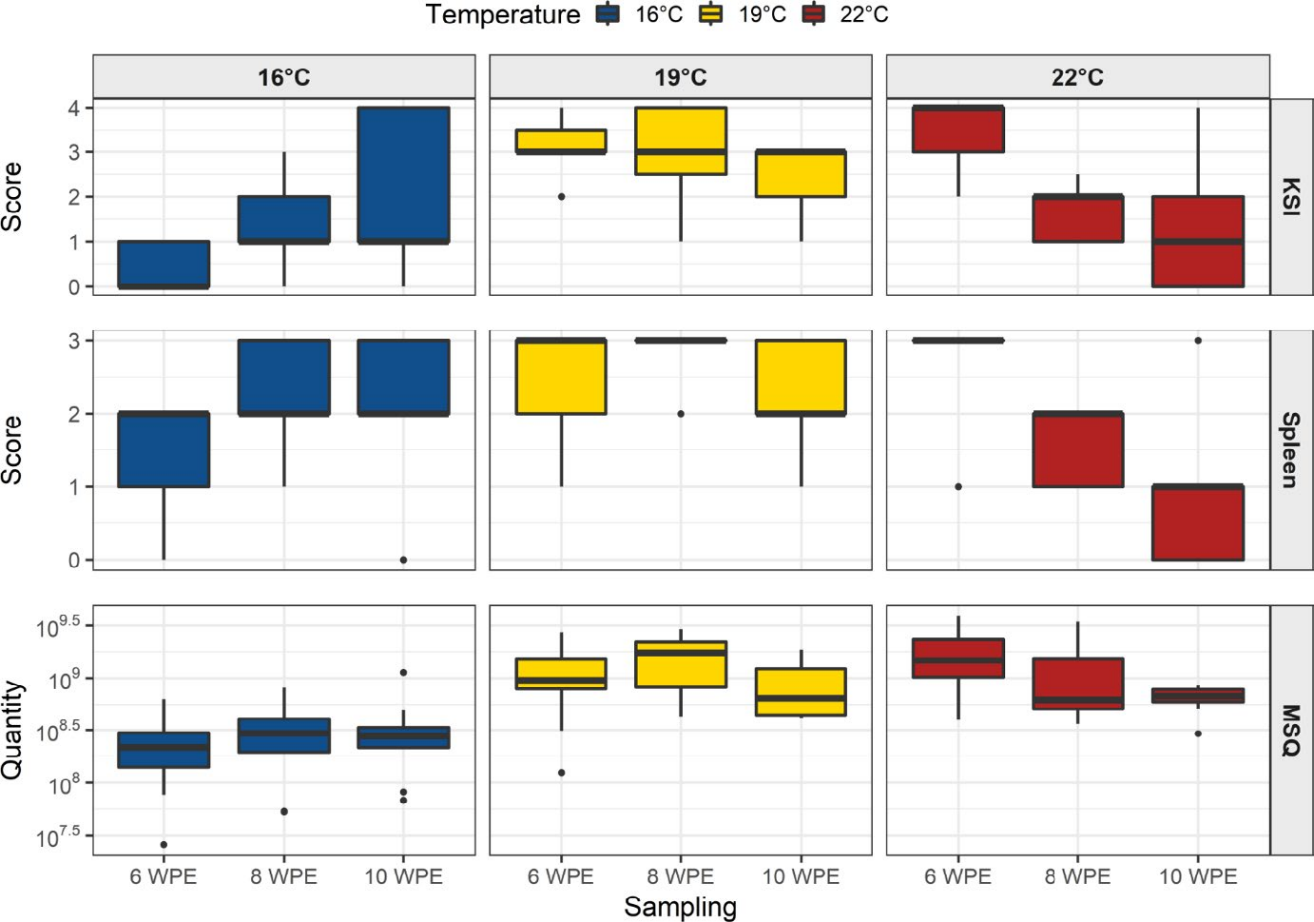
Box plots showing KSI, spleen score and MSQ (mean starting quantity) of sampled infected brown trout (Salmo trutta) at 6, 8 and 10 weeks post‐exposure (WPE). 16°C group in blue, 19°C group in yellow and 22°C group in red colour [Colour figure can be viewed at wileyonlinelibrary.com]

Figure 2 shows the development of KSI along with the spleen scores in sampled infected brown trout from all the water temperature groups at 6, 8 and 10 wpe. The development of the same parameters for killed fish is shown in Figure 1. In the 19°C and 22°C groups, an increase in both KSI and spleen scores was observed with increasing morbidity.

The KSI and spleen scores of the 16°C group increased continuously over the complete sampling period with median values staying at or below 2 for the KSI and for the spleen score. The 19°C group displayed already high values at the first sampling, which remained stable through the second sampling for the KSI and even increased slightly for the spleen score. At 10 wpe, sampling KSI and spleen scores decreased to their lowest with median values at 3 and 2, respectively. In the 22°C group, the overall highest KSI scores were observed at the 6 wpe sampling. Both KSI and spleen scores decreased throughout the whole experiment to reach median values of 1 at 10 wpe.

The examination did not reveal any other parasitical manifestation. The bacteriological examination showed coinfections with *Aeromonas* sp., *Pseudomonas* sp., *Shewanella* sp., *Photobacterium* sp. and *Lelottia* sp. in 5 killed brown trout from the 19°C control group, 3 killed fish from the exposed group, 1 fish from the 22°C and 3 fish from the 22°C exposed group.

### 3.3 Detection and quantification of *T. bryosalmonae* in kidney tissue

All the brown trout that were exposed to *T. bryosalmonae* spores tested positive for the parasite, whereas all the control fish and those tested before the start of the experiment tested negative. Two samples from exposed fish were excluded from analysis because the difference between the Cq values of the duplicates was >1. The MSQ values of killed exposed fish from all the temperature groups were visualized using a LOESS curve shown in Figure 1. The MSQ values of the fish in the 19°C and 22°C temperature groups were increasing faster compared to 16°C group. After 9 wpe, the MSQ values of all the temperature groups reached the same level.

The MSQ values of the sampled infected fish from all the temperature groups are shown in the box plots in Figure 2. The values remained approximately at the same level throughout the experiment in the 16°C group, but in the 19°C and 22°C, they were higher than those in the 16°C group at every sampling time point.

The MSQ values of the 19°C group were already high at 6 wpe and reached its maximum at 8 wpe. After that, MSQ values started to decrease to a level lower than those of the first sampling. For the 22°C group, the MSQ peaked at the first sampling (6 wpe), displaying the overall highest measured values of the experiment. The successive two samplings showed a continuous decline in the MSQ values as well.

## 4 DISCUSSION

Proliferative kidney disease is a widespread parasitic disease of salmonid fish contributing to the decline of wild brown trout populations (Wahli et al., 2007). This study showed that surveyed clinical signs of PKD (KSI, spleen score), parasite burden (MSQ) and PKD‐related morbidity are dependent on water temperature in the brown trout. Compared to the infected brown trout in the 16°C group, the infected fish in the 19°C and 22°C group had significantly increased morbidity rates (Figure 1). In the 16°C group, the cumulative morbidity reached 12.1% after 10 wpe, which is in line with the results of the previous studies, where under laboratory conditions, zero or low mortalities—up to 7%—were observed in PKD‐infected brown trout maintained at 15°C for 70 days (Bailey et al., 2017), 16.5°C for 17 weeks (Kumar et al., 2013) and 15°C for 1 year (Strepparava et al., ,^2018^, ^2020^). The results confirm our hypothesis that clinical signs and morbidity of PKD‐infected fish increase with increasing water temperature. Also, there were no or only minor differences between the cumulative morbidity rates and the clinical signs between the fish in 19°C and 22°C groups, indicating a tipping point in water temperature, from which on there is no significant difference in the survival probability of the brown trout anymore.

A comparison between the cumulative morbidity of brown trout and the rainbow trout in earlier experiments has clarified that rainbow trout are more likely to die from a PKD infection. The cumulative morbidity surveyed in our study was overall lower than the one in an earlier study with rainbow trout (Bettge, Segner, et al., 2009). In total, 45.5% of infected rainbow trout died within 49 dpe at 16°C, while only 12.1% of brown trout died during our experiment. At 19°C, 85% of the exposed rainbow trout died, whereas the morbidity of brown trout in our study was only 69.7% (Bettge, Segner, et al., 2009). The immune response to *T. bryosalmonae* of brown trout has been shown to differ from that of rainbow trout, which may explain the differing morbidity rates between the two species (Bailey et al., 2019; Kumar et al., 2015). Therefore, one should be extremely careful while extrapolating the results from experiments conducted on rainbow trout to other salmonid species such as *S. trutta*. One should be even more cautious in drawing conclusions about developments in natural waters from the results of a laboratory experiment. There are no constant water temperatures in rivers, but rather large diurnal fluctuations and weather‐related temperature differences, the influence of which on the course of PKD has not been researched to date. Therefore, further experiments should focus on field studies to investigate the influence of temperature amplitudes and temperature fluctuations on the course of the disease.

The investigated parameters (KSI, spleen score and MSQ) displayed similar patterns within each of the temperature groups. While the 16°C group exhibited low KSI and spleen score values during the first sampling (6 wpe), consistently increasing levels were recorded during the next two samplings (8 wpe and 10 wpe), indicating no reduction of signs during the observation period. MSQ values reached a plateau at the sampling 8 wpe (Figure 2). These observations are in line with findings from infected brown trout kept at 15°C for 1 year where the kidney pathology increased until 12 wpe and parasite intensity entered the plateau phase at 7 wpe (Strepparava et al., ,^2018^, ^2020^). For the 19°C group, these values reached a peak at 8 wpe and returned to lower levels at the 10 wpe sampling, thereby suggesting a subsequent decrease. The brown trout in the 22°C group experienced the overall highest values already at the 6 wpe sampling, after which the values decreased continuously, indicating an early onset of clinical signs and also a faster decrease in the signs when compared to the other groups.

These findings suggest that higher temperatures lead to a faster progression of the signs and in turn lead to faster recovery. However, the MSQ medians in the 16°C group, at all sampling points, were lower than any of the samplings in the 19°C or 22°C groups, indicating a weaker PKD infection that did not lead to very high KSI and spleen scores during the observation period suggesting a less lethal course of the disease. This is also supported by the fact that the periods of highest morbidity (19°C: 6–10 wpe and 22°C: 3–6 wpe) were observed in the lead up to the sampling time points with the highest scores. These observations are in line with findings from Strepparava et al. who showed that temperature is highly influential on the parasite proliferation rate in the kidney. Once a certain threshold of parasite intensity is reached, spore shedding starts after which temperature's effect on spore excretion is negligible, suggesting that increased temperature acts as an accelerator of disease progression (Strepparava et al., 2018).

However, the 22°C group (where none of the killed fish reached a maximal score after 6 wpe) suggests that once these values peak, they start to decline, leading to a decline in the morbidity as well. Since we did not observe a peak in the values of the observed parameters in the 16°C group, it is possible that the clinical signs would have increased after 10 wpe reaching similarly high KSI, spleen scores and morbidity rates as the 19°C and 22°C groups. In rainbow trout kept at 16°C for 49 days post‐exposure, the KSI scores almost reached levels of the 19°C group (KSI = 2.5) while cumulative mortality remained much lower without reaching a peak (16°C: 45.5%; 19°C: 85%) (Bettge, Segner, et al., 2009). Therefore, an extended duration of the experiment would help in monitoring the decrease in clinical signs until complete recovery is achieved, which might confirm not only a faster onset of clinical signs with higher KSI and spleen scores as well as higher morbidity rates but also a shorter recovery period in the infected brown trout kept at higher water temperatures (19°C, 22°C) compared to lower water temperatures (16°C). Additionally, regular sampling during the early phase of infection is necessary to follow the exact course of clinical signs in fish kept at 19°C and 22°C and also to indicate whether the fish in the 22°C group displayed signs earlier than those in the 19°C group.

Ten fish were killed within 3 wpe, and none of them displayed any signs of kidney enlargement (scores of 0) and only one had a score higher than 0 for the spleen. The MSQ values increased with time post‐exposure, suggesting a PKD‐associated morbidity that is not directly related to macroscopically visible kidney damage. Six of these fish were in the 19°C group, where four of these were from the same tank, further suggesting an additional effect such as bacterial coinfections potentially causing early morbidity.

Another possible influence on mortality rates could be the time when water temperatures of a stream rise above 15°C (Hedrick et al., 1993). If critical temperatures are reached or exceeded early in the year and thus very early developmental stages of brown trout become infected with PKD, the mortality rates could well be much higher than in our study. However, studies on early juvenile life‐history stages (fry, early parr) are still pending. The results of our study underline the importance that climate change will have on PKD. Rising water temperatures in the rivers are reported in the Alpine area, an important habitat of autochthonous brown trout (Filipe et al., 2013; Hari et al., 2006; Melcher et al., 2013). Since the expected rise in temperature may increase the prevalence and severity of PKD (Waldner et al., 2019), it may further stress the wild brown trout populations (Borgwardt et al., 2020) that are already under pressure because of habitat destruction, returning predators like cormorant (*Phalacrocorax carbo*), and otter (*Lutra lutra*), water pollution and incorrectly applied stock management strategies (Jacobsen, 2005; Koed et al., 2006; Pinter et al., 2019; Sittenthaler et al., 2015). Therefore, management of brown trout stocks should consider the interplay of these different factors to effectively protect populations in the future.

## CONFLICT OF INTEREST

We have no conflict of interests to declare.

## ETHICAL APPROVAL

The experiment was conducted according to the relevant guidelines and regulations of §26 of the Austrian Law for Animal Experiments (Tierversuchsgesetz 2012) as well as Directive 2010/63/EU. The institutional ethics committee of the University of Veterinary Medicine, Vienna, Austria, and the national authority approved the experiments under the permission numbers BMWFW GZ 68.205/0124‐V/3b/2018.

## Supporting information

App S1

## Data Availability

There are no additional data available.

## References

[jfd13361-bib-0002] Ahmad, F. , Debes, P. V. , Nousiainen, I. , Kahar, S. , Pukk, L. , Gross, R. , Ozerov, M. , & Vasemägi, A. (2020). The strength and form of natural selection on transcript abundance in the wild. Molecular Ecology. 1–14. 10.1111/mec.15743 33219570 PMC8246785

[jfd13361-bib-0003] Almodóvar, A. , Nicola, G. G. , Ayllón, D. , & Elvira, B. (2012). Global warming threatens the persistence of Mediterranean brown trout. Global Change Biology, 18(5), 1549–1560. 10.1111/j.1365-2486.2011.02608.x

[jfd13361-bib-0004] Anderson, C. L. , Canning, E. U. , & Okamura, B. (1999). Molecular data implicate bryozoans as hosts for (Phylum Myxozoa) and identify a clade of bryozoan parasites within the Myxozoa. Parasitology, 119, 555–561. 10633916 10.1017/s003118209900520x

[jfd13361-bib-0065] APCC (2018). Österreichischer Special Report Gesundheit, Demographie und Klimawandel (ASR18). Austrian Panel on Climate Change (APCC), Verlag der Österreichische Akademie der Wissenschaften, Wien, Österreich, 340 Seiten, ISBN 978‐3‐7001‐8427‐0.

[jfd13361-bib-0005] Bailey, C. , Rubin, A. , Strepparava, N. , Segner, H. , Rubin, J.‐F. , & Wahli, T. (2018). Do fish get wasted? Assessing the influence of effluents on parasitic infection of wild fish. PeerJ, 6, e5956. 10.7717/peerj.5956 30479904 PMC6238765

[jfd13361-bib-0006] Bailey, C. , Schmidt‐Posthaus, H. , Segner, H. , Wahli, T. , & Strepparava, N. (2017). Are brown trout Salmo trutta fario and rainbow trout Oncorhynchus mykiss two of a kind? A comparative study of salmonids to temperature‐influenced *Tetracapsuloides bryosalmonae* infection. Journal of Fish Diseases, 41(2), 191–198. 10.1111/jfd.12694 28914447

[jfd13361-bib-0007] Bailey, C. , Strepparava, N. , Wahli, T. , & Segner, H. (2019). Exploring the immune response, tolerance and resistance in proliferative kidney disease of salmonids. Developmental and Comparative Immunology, 90, 165–175. 10.1016/j.dci.2018.09.015 30248359

[jfd13361-bib-0008] Beraldo, P. , Berton, D. , Giavenni, R. , & Galeotti, M. (2006). First report on proliferative kidney disease (PKD) in marble trout (Salmo trutta marmoratus, Cuvier 1817). Bulletin‐ European Association of Fish Pathologists, 26(3), 143–150.

[jfd13361-bib-0009] Bettge, K. , Segner, H. , Burki, R. , Schmidt‐Posthaus, H. , & Wahli, T. (2009). Proliferative kidney disease (PKD) of rainbow trout: Temperature‐ and time‐related changes of *Tetracapsuloides bryosalmonae* DNA in the kidney. Parasitology, 136(6), 615–625. 10.1017/S0031182009005800 19366483

[jfd13361-bib-0010] Bettge, K. , Wahli, T. , Segner, H. , & Schmidt‐Posthaus, H. (2009). Proliferative kidney disease in rainbow trout: Time‐ and temperature‐related renal pathology and parasite distribution. Diseases of Aquatic Organisms, 83(1), 67–76. 10.3354/dao01989 19301638

[jfd13361-bib-0011] Borgwardt, F. , Unfer, G. , Auer, S. , Waldner, K. , El‐Matbouli, M. , & Bechter, T. (2020). Direct and indirect climate change impacts on brown trout in central Europe: How thermal regimes reinforce physiological stress and support the emergence of diseases. Frontiers in Environmental Science, 8. 1–14. 10.3389/fenvs.2020.00059

[jfd13361-bib-0012] Bruneaux, M. , Visse, M. , Gross, R. , Pukk, L. , Saks, L. , Vasemägi, A. , & Tschirren, B. (2017). Parasite infection and decreased thermal tolerance: Impact of proliferative kidney disease on a wild salmonid fish in the context of climate change. Functional Ecology, 31(1), 216–226. 10.1111/1365-2435.12701

[jfd13361-bib-0013] Carraro, L. , Bertuzzo, E. , Mari, L. , Fontes, I. , Hartikainen, H. , Strepparava, N. , Schmidt‐Posthaus, H. , Wahli, T. , Jokela, J. , Gatto, M. , & Rinaldo, A. (2017). Integrated field, laboratory, and theoretical study of PKD spread in a Swiss prealpine river. Proceedings of the National Academy of Sciences of the United States of America, 114(45), 11992–11997. 10.1073/pnas.1713691114 29078391 PMC5692590

[jfd13361-bib-0014] Carraro, L. , Hartikainen, H. , Jokela, J. , Bertuzzo, E. , & Rinaldo, A. (2018). Estimating species distribution and abundance in river networks using environmental DNA. Proceedings of the National Academy of Sciences of the United States of America, 115(46), 11724–11729. 10.1073/pnas.1813843115 30373831 PMC6243290

[jfd13361-bib-0015] Clifton‐Hadley, R. S. , Bucke, D. , & Richards, R. H. (1987). A study of the sequential clinical and pathological changes during proliferative kidney disease in rainbow trout, Salmo gairdneri Richardson. Journal of Fish Diseases, 10(5), 335–352. 10.1111/j.1365-2761.1987.tb01081.x

[jfd13361-bib-0016] De Kinkelin, P. , Okamura, B. , Hedrick, R. P. , & Baxa, D. V. (2004). Malacosporean‐like spores in urine of rainbow trout react with antibody and DNA probes to *Tetracapsuloides bryosalmonae* . Parasitology Research, 92(1), 81–88. 10.1007/s00436-003-0986-3 14610667

[jfd13361-bib-0017] Debes, P. V. , Gross, R. , & Vasemägi, A. (2017). Quantitative genetic variation in, and environmental effects on, pathogen resistance and temperature‐dependent disease severity in a wild trout. The American Naturalist, 190(2), 244–265. 10.1086/692536 28731797

[jfd13361-bib-0018] Elliott, J. M. , & Elliott, J. A. (2010). Temperature requirements of Atlantic salmon *Salmo salar*, brown trout *Salmo trutta* and Arctic charr *Salvelinus alpinus*: Predicting the effects of climate change. Journal of Fish Biology, 77(8), 1793–1817. 10.1111/j.1095-8649.2010.02762.x 21078091

[jfd13361-bib-0019] El‐Matbouli, M. , & Hoffman, R. W. (2002). Influence of water quality on the outbreak of proliferative kidney disease ‐ field studies and exposure experiments. Journal of Fish Diseases, 25(8), 459–467. 10.1046/j.1365-2761.2002.00393.x

[jfd13361-bib-0020] Ferguson, H. W. , & Needham, E. A. (1978). Proliferative kidney disease in rainbow trout Salmo gairdneri Richardson. Journal of Fish Diseases, 1(1), 91–108. 10.1111/j.1365-2761.1978.tb00008.x

[jfd13361-bib-0021] Filipe, A. F. , Markovic, D. , Pletterbauer, F. , Tisseuil, C. , de Wever, A. , Schmutz, S. , & Freyhof, J. (2013). Forecasting fish distribution along stream networks: Brown trout (*Salmo trutta*) in Europe. Diversity and Distributions, 19(8), 1059–1071. 10.1111/ddi.12086

[jfd13361-bib-0022] Gilad, O. , Yun, S. , Adkison, M. A. , Way, K. , Willits, N. H. , Bercovier, H. , & Hedrick, R. P. (2003). Molecular comparison of isolates of an emerging fish pathogen, koi herpesvirus, and the effect of water temperature on mortality of experimentally infected koi. The Journal of General Virology, 84(Pt 10), 2661–2667. 10.1099/vir.0.19323-0 13679599

[jfd13361-bib-0023] Grabner, D. S. , & El‐Matbouli, M. (2008). Transmission of *Tetracapsuloides bryosalmonae* (Myxozoa: Malacosporea) to Fredericella sultana (Bryozoa: Phylactolaemata) by various fish species. Diseases of Aquatic Organisms, 79(2), 133–139. 10.3354/dao01894 18500029

[jfd13361-bib-0024] Grabner, D. S. , & El‐Matbouli, M. (2009). Comparison of the susceptibility of brown trout (*Salmo trutta*) and four rainbow trout (*Oncorhynchus mykiss*) strains to the myxozoan *Tetracapsuloides bryosalmonae*, the causative agent of proliferative kidney disease (PKD). Veterinary Parasitology, 165(3–4), 200–206. 10.1016/j.vetpar.2009.07.028 19683396

[jfd13361-bib-0025] Grabner, D. S. , & El‐Matbouli, M. (2010). *Tetracapsuloides bryosalmonae* (Myxozoa: Malacosporea) portal of entry into the fish host. Diseases of Aquatic Organisms, 90(3), 197–206. 10.3354/dao02236 20815328

[jfd13361-bib-0026] Hari, R. E. , Livingstone, D. M. , Siber, R. , Burkhardt‐Holm, P. , & Guttinger, H. (2006). Consequences of climatic change for water temperature and brown trout populations in Alpine rivers and streams. Global Change Biology, 12(1), 10–26. 10.1111/j.1365-2486.2005.001051.x

[jfd13361-bib-0027] Hartikainen, H. , Gruhl, A. , & Okamura, B. (2014). Diversification and repeated morphological transitions in endoparasitic cnidarians (Myxozoa: Malacosporea). Molecular Phylogenetics and Evolution, 76, 261–269. 10.1016/j.ympev.2014.03.010 24675700

[jfd13361-bib-0028] Hartikainen, H. , Johnes, P. , Moncrieff, C. , & Okamura, B. (2009). Bryozoan populations reflect nutrient enrichment and productivity gradients in rivers. Freshwater Biology, 54(11), 2320–2334. 10.1111/j.1365-2427.2009.02262.x

[jfd13361-bib-0029] Hedrick, R. P. , MacConnell, E. , & de Kinkelin, P. (1993). Proliferative kidney disease of salmonid fish. Annual Review of Fish Diseases, 3, 277–290. 10.1016/0959-8030(93)90039-E

[jfd13361-bib-0030] Holzer, A. S. , Sommerville, C. , & Wootten, R. (2006). Molecular studies on the seasonal occurrence and development of five myxozoans in farmed *Salmo trutta* L. Parasitology, 132(Pt 2), 193–205. 10.1017/S0031182005008917 16216135

[jfd13361-bib-0031] IPCC (2018). Global warming of 1.5°C: An IPCC Special Report on the impacts of global warming of 1.5°C above pre‐industrial levels and related global greenhouse gas emission pathways, in the context of strengthening the global response to the threat of climate change, sustainable development, and efforts to eradicate poverty. Retrieved from https://www.ipcc.ch/site/assets/uploads/2018/10/SR15_SPM_version_stand_alone_LR.pdf

[jfd13361-bib-0032] Isaak, D. J. , Young, M. K. , Luce, C. H. , Hostetler, S. W. , Wenger, S. J. , Peterson, E. E. , Ver Hoef, J. M. , Groce, M. C. , Horan, D. L. , & Nagel, D. E. (2016). Slow climate velocities of mountain streams portend their role as refugia for cold‐water biodiversity. Proceedings of the National Academy of Sciences of the United States of America, 113(16), 4374–4379. 10.1073/pnas.1522429113 27044091 PMC4843441

[jfd13361-bib-0033] Jacobsen, L. (2005). Otter (*Lutra lutra*) predation on stocked brown trout (*Salmo trutta*) in two Danish lowland rivers. Ecology of Freshwater Fish, 14(1), 59–68. 10.1111/j.1600-0633.2004.00076.x

[jfd13361-bib-0034] Jencic, V. , Zajc, U. , Kusar, D. , Ocepek, M. , & Pate, M. (2014). A survey on *Tetracapsuloides bryosalmonae* infections in Slovene fresh waters. Journal of Fish Diseases, 37(8), 711–717. 10.1111/jfd.12160 23941273

[jfd13361-bib-0035] Karvonen, A. , Rintamäki, P. , Jokela, J. , & Valtonen, E. T. (2010). Increasing water temperature and disease risks in aquatic systems: Climate change increases the risk of some, but not all, diseases. International Journal for Parasitology, 40(13), 1483–1488. 10.1016/j.ijpara.2010.04.015 20580904

[jfd13361-bib-0036] Kent, M. L. , Khattra, J. , Hervio, D. M. L. , & Devlin, R. H. (1998). Ribosomal DNA sequence analysis of isolates of the PKX Myxosporean and their relationship to members of the genus Sphaerospora. Journal of Aquatic Animal Health, 10(1), 12–21.

[jfd13361-bib-0037] Koed, A. , Baktoft, H. , & Bak, B. D. (2006). Causes of mortality of Atlantic salmon (*Salmo salar*) and brown trout (*Salmo trutta*) smolts in a restored river and its estuary. River Research and Applications, 22(1), 69–78. 10.1002/rra.894

[jfd13361-bib-0038] Kristmundsson, A. , Antonsson, T. , & Arnason, E. (2010). First record of proliferative kidney disease in Iceland. Bulletin of the European Association of Fish Pathologists, 30(1), 35–40.

[jfd13361-bib-0039] Kromp‐Kolb, H. , Lindenthal, T. , & Bohunovsky, L. (2014). Österreichischer Sachstandsbericht Klimawandel 2014. GAIA ‐ Ecological Perspectives for Science and Society, 23(4), 363–365.

[jfd13361-bib-0040] Kumar, G. , Abd‐Elfattah, A. , & El‐Matbouli, M. (2015). Identification of differentially expressed genes of brown trout (*Salmo trutta*) and rainbow trout (*Oncorhynchus mykiss*) in response to *Tetracapsuloides bryosalmonae* (Myxozoa). Parasitology Research, 114(3), 929–939. 10.1007/s00436-014-4258-1 25563603 PMC4336411

[jfd13361-bib-0041] Kumar, G. , Abd‐Elfattah, A. , Saleh, M. , & El‐Matbouli, M. (2013). Fate of *Tetracapsuloides bryosalmonae* (Myxozoa) after infection of brown trout *Salmo trutta* and rainbow trout *Oncorhynchus mykiss* . Diseases of Aquatic Organisms, 107(1), 9–18. 10.3354/dao02665 24270019 PMC3962845

[jfd13361-bib-0042] Le Morvan, C. , Troutaud, D. , & Deschaux, P. (1998). Differential effects of temperature on specific and nonspecific immune defences in fish. The Journal of Experimental Biology, 201(Pt 2), 165–168. 9405298 10.1242/jeb.201.2.165

[jfd13361-bib-0043] Lewisch, E. , Unfer, G. , Pinter, K. , Bechter, T. , & El‐Matbouli, M. (2018). Distribution and prevalence of *T. Bryosalmonae* in Austria: A first survey of trout from rivers with a shrinking population. Journal of Fish Diseases, 41(10), 1549–1557.Advance Online Publication. 30027582 10.1111/jfd.12863

[jfd13361-bib-0044] Longshaw, M. , Feist, S. W. , Canning, E. U. , & Okamura, B. (1999). First identification of PKX in bryozoans from the United Kingdom – molecular evidence. Bulletin of the European Association of Fish Pathologists, 19(4), 146–149.

[jfd13361-bib-0045] Longshaw, M. , Le Deuff, R.‐M. , Harris, A. F. , & Feist, S. W. (2002). Development of proliferative kidney disease in rainbow trout, *Oncorhynchus mykiss* (Walbaum), following short‐term exposure to *Tetracapsula bryosalmonae* infected bryozoans. Journal of Fish Diseases, 25(8), 443–449. 10.1046/j.1365-2761.2002.00353.x

[jfd13361-bib-0046] Markovic, D. , Scharfenberger, U. , Schmutz, S. , Pletterbauer, F. , & Wolter, C. (2013). Variability and alterations of water temperatures across the Elbe and Danube River Basins. Climatic Change, 119(2), 375–389. 10.1007/s10584-013-0725-4

[jfd13361-bib-0047] Melcher, A. , Pletterbauer, F. , Kremser, H. , & Schmutz, S. (2013). Temperaturansprüche und Auswirkungen des Klimawandels auf die Fischfauna in Flüssen und unterhalb von Seen. Österreichische Wasser‐ Und Abfallwirtschaft, 65(11–12), 408–417. 10.1007/s00506-013-0118-y

[jfd13361-bib-0048] Okamura, B. , Gruhl, A. , & Bartholomew, J. (Eds.) (2015). Myxozoan evolution, ecology and development. Springer. 10.1007/978-3-319-14753-6

[jfd13361-bib-0049] Palikova, M. , Papezikova, I. , Markova, Z. , Navratil, S. , Mares, J. , Mares, L. , Vojtek, L. , Hyrsl, P. , Jelinkova, E. , & Schmidt‐Posthaus, H. (2017). Proliferative kidney disease in rainbow trout (*Oncorhynchus mykiss*) under intensive breeding conditions: Pathogenesis and haematological and immune parameters. Veterinary Parasitology, 238, 5–16. 10.1016/j.vetpar.2017.03.003 28291603

[jfd13361-bib-0050] Peeler, E. J. , Feist, S. W. , Longshaw, M. , Thrush, M. A. , & St‐Hilaire, S. (2008). An assessment of the variation in the prevalence of renal myxosporidiosis and hepatitis in wild brown trout, *Salmo trutta* L., within and between rivers in South‐West England. Journal of Fish Diseases, 31(10), 719–728. 10.1111/j.1365-2761.2008.00942.x 18681903

[jfd13361-bib-0051] Pinter, K. , Epifanio, J. , & Unfer, G. (2019). Release of hatchery‐reared brown trout (*Salmo trutta*) as a threat to wild populations? A case study from Austria. Fisheries Research, 219, 105296. 10.1016/j.fishres.2019.05.013

[jfd13361-bib-0052] Pletterbauer, F. , Graf, W. , & Schmutz, S. (2016). Effect of biotic dependencies in species distribution models: The future distribution of *Thymallus thymallus* under consideration of *Allogamus auricollis* . Ecological Modelling, 327, 95–104. 10.1016/j.ecolmodel.2016.01.010

[jfd13361-bib-0053] R Core Team (2020). R: A language and environment for statistical computing. Retrieved from https://www.R‐project.org/

[jfd13361-bib-0054] Schmidt‐Posthaus, H. , Hirschi, R. , & Schneider, E. (2015). Proliferative kidney disease in brown trout: Infection level, pathology and mortality under field conditions. Diseases of Aquatic Organisms, 114(2), 139–146. 10.3354/dao02855 25993888

[jfd13361-bib-0055] Sittenthaler, M. , Bayerl, H. , Unfer, G. , Kuehn, R. , & Parz‐Gollner, R. (2015). Impact of fish stocking on Eurasian otter (*Lutra lutra*) densities: A case study on two salmonid streams. Mammalian Biology ‐ Zeitschrift Für Säugetierkunde, 80(2), 106–113. 10.1016/j.mambio.2015.01.004

[jfd13361-bib-0056] Stauffer, J. , Bruneaux, M. , Panda, B. , Visse, M. , Vasemägi, A. , & Ilmonen, P. (2017). Telomere length and antioxidant defense associate with parasite‐induced retarded growth in wild brown trout. Oecologia, 185(3), 365–374. 10.1007/s00442-017-3953-x 28900791

[jfd13361-bib-0057] Strepparava, N. , Ros, A. , Hartikainen, H. , Schmidt‐Posthaus, H. , Wahli, T. , Segner, H. , & Bailey, C. (2020). Effects of parasite concentrations on infection dynamics and proliferative kidney disease pathogenesis in brown trout (*Salmo trutta*). Transboundary and Emerging Diseases, 67(6), 2642–2652. 10.1111/tbed.13615 32386103

[jfd13361-bib-0058] Strepparava, N. , Segner, H. , Ros, A. , Hartikainen, H. , Schmidt‐posthaus, H. , & Wahli, T. (2018). Temperature‐related parasite infection dynamics: The case of proliferative kidney disease of brown trout. Parasitology, 145(3), 281–291. 10.1017/S0031182017001482 28831940

[jfd13361-bib-0059] Tops, S. , Hartikainen, H.‐L. , & Okamura, B. (2009). The effects of infection by *Tetracapsuloides bryosalmonae* (Myxozoa) and temperature on Fredericella sultana (Bryozoa). International Journal for Parasitology, 39(9), 1003–1010. 10.1016/j.ijpara.2009.01.007 19504757

[jfd13361-bib-0060] Tops, S. , Lockwood, W. , & Okamura, B. (2006). Temperature‐driven proliferation of *Tetracapsuloides bryosalmonae* in bryozoan hosts portends salmonid declines. Diseases of Aquatic Organisms, 70(3), 227–236. 10.3354/dao070227 16903234

[jfd13361-bib-0061] Unfer, G. , Holzer, G. , Gallowitsch, M. , Gumpinger, C. , Hundlinger, R. , & El‐Matbouli, M. (2015). Ausbruch der PKD (Proliferative Kidney Disease) im Kamp im Sommer 2014: Ein Ereignisbericht, der nicht ohne Konsequenzen bleiben darf!. Österreichs Fischerei, 04, 104–108.

[jfd13361-bib-0062] Wahli, T. , Bernet, D. , Steiner, P. A. , & Schmidt‐Posthaus, H. (2007). Geographic distribution of *Tetracapsuloides bryosalmonae* infected fish in Swiss rivers: An update. Aquatic Sciences, 69(1), 3–10. 10.1007/s00027-006-0843-4

[jfd13361-bib-0063] Waldner, K. , Bechter, T. , Auer, S. , Borgwardt, F. , El‐Matbouli, M. , & Unfer, G. (2019). A brown trout (*Salmo trutta*) population faces devastating consequences due to proliferative kidney disease and temperature increase: A case study from Austria. Ecology of Freshwater Fish, 29, 465–476.Advance online publication.

[jfd13361-bib-0064] Wickham, H. (2016). ggplot2: Elegant graphics for data analysis. Springer‐Verlag.

