# Supplementary material for: Effect of water temperature on the morbidity of Tetracapsuloides bryosalmonae (Myxozoa) to brown trout (Salmo trutta) under laboratory conditions
Source: J Fish Dis. 2021 Mar 3;44(7):1005–13. doi: 10.1111/jfd.13361 (PMC8248319; doi:10.1111/jfd.13361)
Supplement: Supplementary file 1 — App S1 [file JFD-44-1005-s001.docx]

Appendix S1

| **Score sheet for evaluation of endpoints of experimental fish** | |  |
| --- | --- | --- |
| **General Indicators** | **Scoring of independent variables** | **Individual score** |
| Feed consumption | 0. normal |  |
|  | 1. reduced |  |
|  | 2. no intake |  |
| Swimming pattern | 0. normal |  |
|  | 1. intermittent loss of equilibrium |  |
|  | 2. frequent loss of equilibrium |  |
|  | 3. complete loss of equilibrium |  |
| Colour change | 0. normal |  |
|  | 1. slightly dark/pale |  |
|  | 2. intense dark/pale |  |
| Grouping | 0. normal |  |
|  | 2. separating |  |
| Respiratory rate | 0. normal |  |
|  | 1. mildly enforced/reduced |  |
|  | 2. moderately enforced/reduced |  |
|  | 3. highly enforced/reduced |  |
| **Specific indicators (infectious diseases)** |  |  |
| Skin and fin condition | 0. normal |  |
|  | 1. mild changes* |  |
|  | 2. moderate changes* |  |
|  | 3.severe changes* |  |
| Eyes | 0. normal |  |
|  | 1.mild exophthalmia |  |
|  | 2. moderate exophthalmia |  |
|  | 3. severe exophthalmia |  |
| Abdominal distension | 0.not existing |  |
|  | 1. mild |  |
|  | 2. moderate |  |
|  | 3. severe |  |
| **Total score** |  |  |
|  |  |  |

*e.g. mucus production, haemorrhages, ulcers, fin lesions

|  | **Score key** |
| --- | --- |
| 0 | no action required |
| 1-4 | monitor 3 times a day |
| 5-8 | inform vet |
| >8 | euthanize |
| 3 in any category | euthanize |
